# Integrated transcriptomic and transgenic analyses reveal potential mechanisms of poplar resistance to *Alternaria alternata* infection

**DOI:** 10.1186/s12870-022-03793-5

**Published:** 2022-08-25

**Authors:** Ying Huang, Huijun Ma, Yuanzhi Yue, Tianchang Zhou, Zhenyu Zhu, Chao Wang

**Affiliations:** State Key Laboratory of Tree Genetics and Breeding (Northeast Forestry University), 26 Hexing Road, Harbin, 150040 China

**Keywords:** RNA-Seq, Poplar, *Alternaria alternate*, Defense response, Lipoxygenase gene

## Abstract

**Background:**

*Populus davidiana* × *P. bollena* is a species of poplar from northeastern China that is characterized by cold resistance and fast growth but now suffers from pathogen infections. Leaf blight caused by *Alternaria alternata* has become a common poplar disease that causes serious economic impacts, but the molecular mechanisms of resistance to *A. alternata* in *P. davidiana* × *P. bollena* are still unclear.

**Results:**

In this study, the transcriptomic response of *P. davidiana* × *P. bollena* to *A. alternata* infection was determined via RNA-Seq. Twelve cDNA libraries were generated from RNA isolated from three biological replicates at four time points (0, 2, 3, and 4 d post inoculation), and a total of 5,930 differentially expressed genes (DEGs) were detected (| log_2_ fold change |≥ 1 and FDR values < 0.05). Functional analysis revealed that the DEGs were mainly enriched for the “plant hormone signal transduction” pathway, followed by the “phenylpropanoid biosynthesis” pathway. In addition, DEGs that encode defense-related proteins and are related to ROS metabolism were also identified. Numerous transcription factors, such as the bHLH, WRKY and MYB families, were also induced by *A. alternata* infection. Among these DEGs, those related to JA biosynthesis and JA signal transduction were consistently activated. Therefore, the lipoxygenase gene *PdbLOX2*, which is involved in JA biosynthesis, was selected for functional characterization. Overexpression of *PdbLOX2* enhanced the resistance of *P. davidiana* × *P. bollena* to *A. alternata,* whereas silencing this gene enhanced susceptibility to *A. alternata* infection.

**Conclusions:**

These results provide new insight into the molecular mechanisms of poplar resistance to *A. alternata* infection and provide candidate genes for breeding resistant cultivars using genetic engineering.

**Supplementary Information:**

The online version contains supplementary material available at 10.1186/s12870-022-03793-5.

## Background

To ward off pathogens, plants have developed a complex defense system, which is generally divided into pattern-triggered immunity (PTI) and effector-triggered immunity (ETI), depending on the pathogen molecule recognized [1]. Generally, the PTI can be activated by pathogen-associated molecular patterns (PAMPs), resulting in the accumulation of reactive oxygen species (ROS), activation of mitogen-activated protein kinase (MAPK) cascades and Ca^2+^ signals, transcription of immunity-related genes, as well as secondary metabolites accumulation [2–4]. Among these the secondary metabolites, such as phenolics, alkaloids, sesquiterpenes, oxylipins, saponins, polyamines, and isoflavonoids, contribute to resistance against pathogen infection via preformed or induced physical barriers or chemical barriers [5–7]. Moreover, other signaling molecules, including salicylic acid (SA), jasmonic acid (JA), and ethylene (ET) were also activated in PTI, and these phytohormones act as signals to trigger and mediate a diverse array of defense responses, while other hormones, such as auxin, gibberellic acids, brassinosteroids, and abscisic acid, are also actively involved in plant immunity by feeding into the SA-JA-ET backbone of the immune signaling circuitry [8–10]. To counteract PTI, the microbes secrete effector proteins, which can be recognized by plant resistance (R) proteins, and then lead to activation of ETI in plant [11, 12]. The ETI was characterized by the induction of SA, defense gene transcription, as well as the hypersensitive response (HR), among which the R genes, including the CC-NBS-LRR genes (CNL genes) and TIR-NBS-LRR genes (TNL genes), play important roles, such as help recognize pathogenic effectors or mimic pathogen targets and so on [13–16]. Additionally, transcription factors (TFs) also play roles in both PTI and ETI, including the basal expression of resistance components, the direct TF activity of receptor proteins, and activation downstream of receptor initiation [17–19]. In general, plant defense mechanisms are complex, and there are various ways to deal with different pathogens. Therefore, it is necessary to study the mechanisms that each type of plant uses against infection by different pathogens to provide a theoretical basis for improving the resistance of plants to disease and selecting disease resistance genes. Using RNA-seq, people can characterize the dynamic patterns of genome-wide gene expression during plant–pathogen interactions and help define gene and protein functions. Recently, pathogen-responsive transcriptomes have been studied in many crops and other economically important plants, such as *Citrus jambhiri* [20] apple [21, 22], tomato [23, 24], *Zea mays* [25] and *Nicotiana benthamiana* [26]. However, research on poplar-pathogen interactions has mainly focused on poplar-rust [27, 28], and little is known about the defense mechanisms that poplars use against other pathogens.

*Alternaria* is a cosmopolitan fungal genus whose members commonly act as plant pathogens and infest a wide range of plants, including cereal crops, vegetables and fruits, leading to necrosis and defoliation on leaves, necrosis on fruits and new shoots, a shortened growth cycle, and postharvest decay [29]. To date, methods of controlling *Alternaria* rely mainly on chemical pesticides such as mancozeb, daconil and captafol, but the long-term use of fungicides results in resistance to chemical fungicides and environmental pollution [30]. Thus, an improved understanding of plant defense mechanisms in response to *Alternaria* may help to design safer control strategies and aid in the development of resistant cultivars. To date, several transcriptome studies in different plant species have been performed to understand the mechanisms of the plant defense response to *Alternaria* sp. infection. In chrysanthemum, the reactive oxygen species (ROS), Ca^2+^ and SA/JA or ET signaling pathways are induced to defend against *Alternaria* sp. [31, 32]. ET-/H_2_O_2_-mediated programmed cell death (PCD) and detoxifying processes have been shown to play vital roles in the interaction between pear and *A. alternata* [33]. Moreover, pathogenesis-related (PRs) genes and cell wall reinforcement-related genes were both downregulated during infection, indicating the susceptibility to *A*. *alternata* of apple “Starking Delicious” may be due to the vulnerability in its cell wall defense and the downregulation of PR proteins [21]. These investigations showed complex, varied interactions between the host and *Alternaria* sp. Poplar (*Populus* spp.), a fast-growing, high-yield tree genus, is vital to the world’s ecological and socioeconomic well-being. Poplar leaf blight caused by *A. alternata* has become a common disease that has serious economic impacts in China [34, 35]. Nevertheless, no detailed analysis has been performed to elucidate the molecular regulatory mechanisms underlying the response of poplar to *A. alternata* infection. In this study, we performed a global transcription analysis of *P. davidiana* × *P. bollena* in response to *A. alternata* infection using RNA-Seq technology, and the differentially expressed genes (DEGs) and significantly enriched pathways associated with pathogen resistance were explored. Multiple potential candidate genes involved in resistance to *A. alternata* infection were also identified, and the function of the lipoxygenase gene *PdbLOX2* was characterized in transgenic poplar. These results provide new insight into the molecular mechanisms of poplar resistance to *A. alternata* infection and will facilitate the breeding of durable, broad-spectrum disease-resistant poplar cultivars in the future.

## Results

### Symptoms and physiological changes in leaves after *Alternaria alternata* infection

**Fig. 1:**
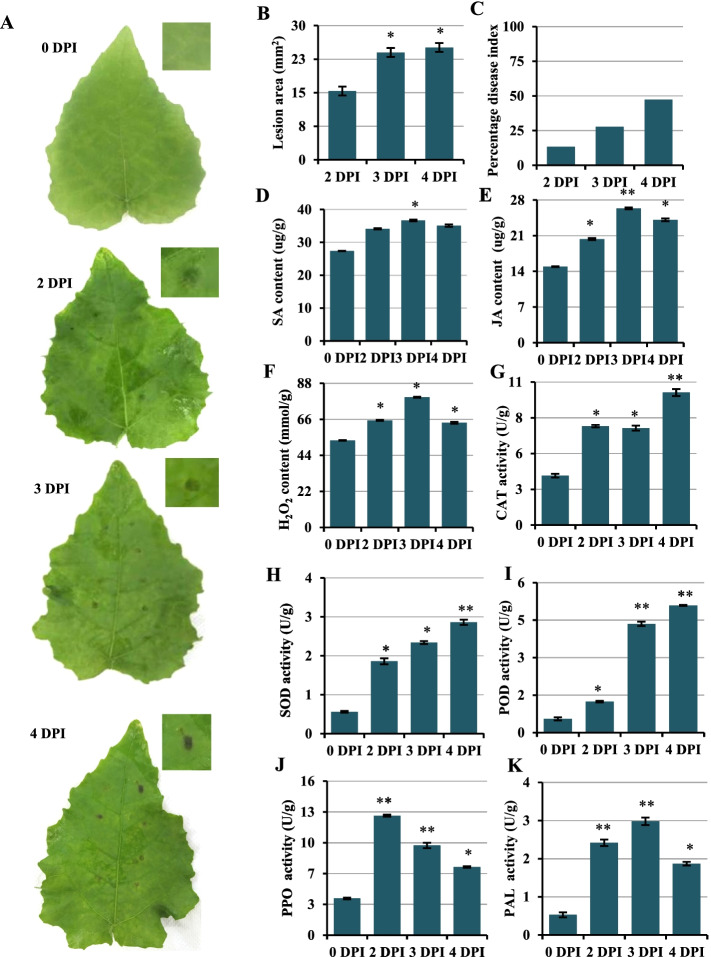
Changes in phenotype and biochemical characteristics during infection. **A** Changes in symptoms in *P*. *davidiana* × *P. bollena* leaves after infection with *A. alternata*. Images show the disease blotches at 0, 2, 3, and 4 DPI, with enlarged views of portions of infected tissue in the inset. **B** Lesion areas after infection. **C** Percentage disease index at 2, 3, and 4 DPI. **D**-**F** Contents of SA (**D**), JA (**E**) and H_2_O_2_ (**F**) in *P*. *davidiana* × *P. bollena* during different stages of infection. **G**-**K** Activities of CAT, SOD, POD, PPO and PAL during infection. The error bar represents the standard deviation of triplicate assays. Asterisks show significant differences between the inoculated and noninoculated leaves (**P < 0.01 and *P < 0. 05)

The progression of disease on inoculated leaves was observed (Fig. 1A-C). The inoculation areas appeared to be water-soaked by 2 DPI, which were hardly obvious. And the blotches comprising damaged tissue appeared to be weakly brown and the blotch size was increased by 3 DPI. Then, over the subsequent 24 h, observations confirmed that the blotch size was no further increased, but the color deepened further. To determine whether the hormones and ROS in the leaves of *P. davidiana* × *P. bollena* responded to *A. alternata*, the contents of SA, JA and hydrogen peroxide (H_2_O_2_) were determined. The results showed that the contents of SA, JA and H_2_O_2_ in inoculated leaves were increased, among which the JA contents were significantly increased compared to the controls during infection (Fig. 1D-F). Additionally, assays of enzymes involved in plant resistance to pathogen infection, such as POD, SOD, PPO, PAL and CAT, were also performed, and the activities of all these enzymes were significantly higher than in the control (Fig. 1G-K). These data indicate that the defense system in *P. davidiana* × *P. bollena* is responsive to *A. alternata* infection.

### RNA-Seq and identification of differentially expressed genes (DEGs) during disease progression

Based on macroscopic observations, four time points, 0 d, 2 d, 3 d and 4 d, were chosen, and 3 biological replicates from those points were then analyzed on an Illumina HiSeq 2500 platform. All together, 12 cDNA libraries were constructed and a total of 58.1 GB of clean reads were generated. The average Q20 and Q30 values of the raw reads were 97.17% and 92.23%, respectively. Approximately 72.13% of the reads were mapped to the reference genome sequences (Table S1).

**Fig. 2:**
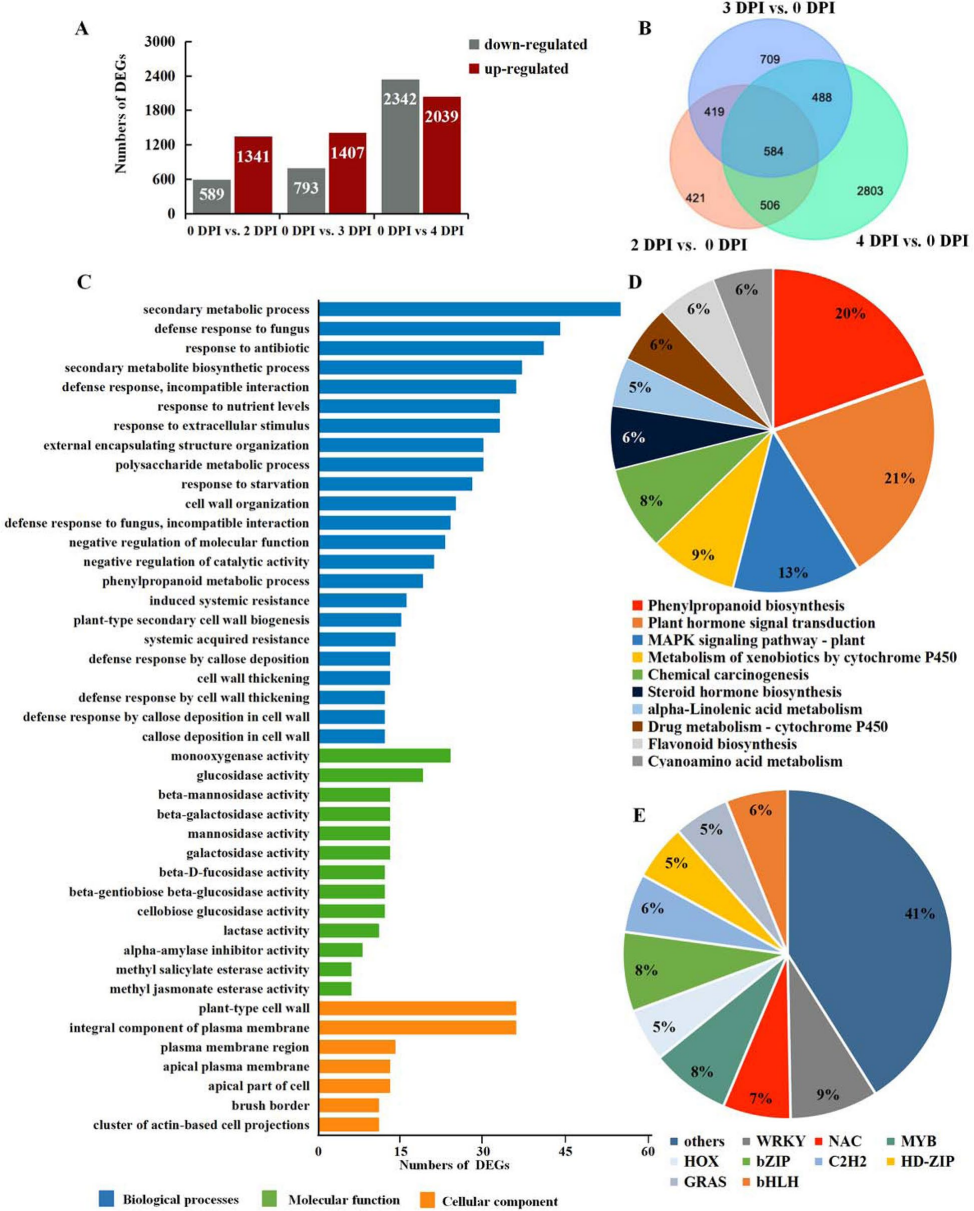
DEGs between samples and functional annotation. **A** Numbers of DEGs between two samples (i.e., 0 vs. 2 DPI, 0 vs. 3 DPI, 0 vs. 4 DPI). DEGs are shown in red (upregulated) and gray (downregulated). **B** Venn diagram of the DEGs in poplar leaves after inoculation with *A. alternata*. **C** GO functional enrichment analysis of DEGs. **D** KEGG pathway enrichment analysis of DEGs. **E** Identification of differentially expressed transcription factors

To investigate the DEGs during pathogenic infection in poplar, the DESeq2 R package was used to analyze and identify genes with expression changes of more than onefold and significance levels of P ≤ 1e − 4. Gene expression was compared between the pathogen-infected and control samples; a comparison of the 2 and 0 DPI libraries identified 1930 DEGs, of which 1341 were upregulated and 589 were downregulated; a comparison of 3 and 0 DPI generated 2200 DEGs with 1407 upregulated and 793 downregulated. Of all the comparisons, the most DEGs, 4381, were found when the 4 and 0 DPI libraries were compared; in that analysis, 2039 were upregulated and 2342 were downregulated, suggesting that this time is particularly important for the poplar in the response to *A. alternata* infection (Fig. 2A). Then, the results above were illustrated as a Venn diagram, both unique and shared DEGs occur between and among pairs (Fig. 2B). For example, 421 DEGs (21.81% of the total) in 2 DPI vs. 0 DPI, 709 DEGs (32.22% of the total) in 3 vs. 0 DPI and 2,803 DEGs (63.98% of the total) in 4 vs. 0 DPI were differentially expressed only in their libraries. In addition, 584 DEGs were shared across all comparisons. These results above suggest that more genes become involved in the defense response during the pathogen infection progresses, and different genes may be involved in the defense response at different infection progresses.

### Validation of RNA-Seq data using RT-qPCR

To verify the accuracy and reproducibility of the RNA-Seq data, the transcript of 20 randomly selected DEGs were analyzed by RT-qPCR. The expression of these genes was in accordance with the RNA-Seq results and with a high correlation coefficient (*R*^2^ = 0.969, *P* < 0.05), demonstrating the reliability of our RNA-Seq data (Fig. S1).

### GO enrichment and KEGG pathway analysis of DEGs

The functional categories of the DEGs induced by pathogenic infection were obtained by GO enrichment and KEGG analysis. The GO enrichment analysis was performed via eggNOG-mapper (Fisher’s exact test, *P* value ≤ 0.05). The most significant enriched biological processes (BPs) were “secondary metabolic process” and “defense response to fungus”. In contrast, the most significant cellular component (CC) terms were related to “plant-type cell wall” and “integral component of plasma membrane”, while molecular function (MF) enrichments were related to “beta-glucosidase activity” and “glucosidase activity” (Fig. 2C). The pathways that displayed significant changes (*P* value ≤ 0.05) were identified via the KEGG database. A total of 17 KEGG pathways were significantly enriched, among which “plant hormone signal transduction” (ko04075), “phenylpropanoid biosynthesis” (ko00940), “MAPK signaling pathway-plant” (ko04016) and “flavonoid biosynthesis” (ko00941) were the most highly represented. Both “plant hormone signal transduction” (ko04075) and “phenylpropanoid biosynthesis” (ko00940) exhibited many DEGs, suggesting that in poplar, plant hormones and phenylpropanoid compounds play significant roles in resistance to pathogen infection. The “MAPK signaling pathway” (ko04016) exhibited the third largest number of DEGs, indicating that during pathogen infection, the expression of internal genes is regulated by various signaling substances in poplar (Fig. 2D). Moreover, the disease resistance genes involved in the “plant–pathogen interaction pathway” (ko04626) or “ras signaling pathway” (ko04014), such as *RPM*, *RGA* and *DRL,* were mainly upregulated after pathogen infection (Fig. S2). Taken together, these results suggest that poplar has evolved a range of different molecular defense strategies depending on the infection stage of the pathogen. Additionally, among these DEGs, 380 TFs that could be divided into 34 TF families were identified, such as the bHLH, MYB, NAC, bZIP (basic leucine zipper), WRKY and HSF families (Table S2). Some of these TFs have previously been reported to be closely related to the plant resistance response to biotic stress. For example, the largest group of pathogen-induced TFs belongs to the WRKY family, which is well known for plant defense, and several MYB, NAC, and bHLH family members were also induced during infection (Fig. 2E). This result suggests that TFs also play roles in the resistance of *P. davidiana* × *P. bollena* to *A. alternata* infection.

### DEGs involved in the phenylpropanoid pathway

**Fig. 3:**
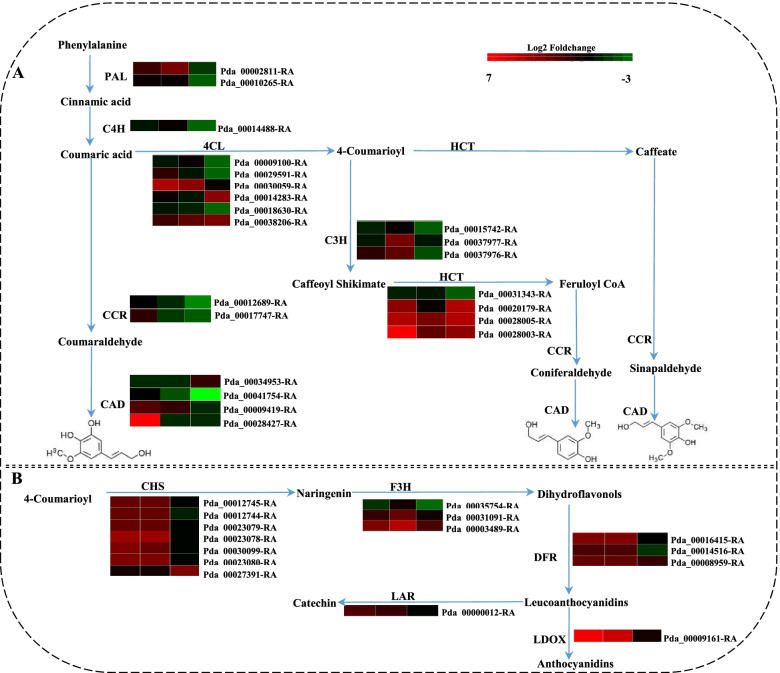
Expression patterns of genes related to the phenylpropane biosynthetic pathway during *A. alternata* infection. **A** The synthesis of lignin. **B** The synthesis of anthocyanin. The log_2_ fold change was colored using Cluster 3.0 (red for upregulated, green for downregulated), each horizontal row represents a DEG with its gene ID, and the vertical columns represent 2, 3, and 4 DPI from left to right

Secondary metabolites are known to be involved in plant defense against pathogens by forming physical or chemical barriers. In this study, DEGs related to secondary metabolism showed significant changes during pathogen infection, especially changes in “phenylpropanoid biosynthesis” (ko00940) and “flavonoid biosynthesis” (ko00941). In total, over one hundred DEGs with significant expression involved in 32 different biosynthetic pathways were enriched in these pathways. A gene for phenylalanine ammonia-lyase (*PAL*) (Pda_00002811-RA), which is involved in the first step of phenylpropanoid biosynthesis and catalyzes lignin accumulation, was upregulated at 3 DPI. Other genes involved in lignin biosynthesis, such as the 4-coumarate-CoA ligase (*4CL*) and shikimate O-hydroxycinnamoyltransferase (*HCT*) genes, were upregulated, while the trans-cinnamate 4-monooxygenase (*C4H*) gene was downregulated. Key genes leading to lignin formation, including p-coumarate 3-hydroxylase (*C3H*), cinnamyl alcohol dehydrogenase (*CAD*) and cinnamoyl-CoA reductase (*CCR*), were up- or downregulated at different time points in this study (Fig. 3A). Moreover, genes for chalcone synthases (*CHS*), a branch point enzyme in flavonoid biosynthesis, were upregulated during pathogen infection, while flavanone 3-hydroxylase (*F3H*), which leads to the formation of quercetin derivatives, was downregulated or upregulated. Additionally, genes encoding leucoanthocyanidin dioxygenase (*LDOX*) and leucoanthocyanidin reductase (*LAR*), which are involved in the synthesis and extension of proanthocyanidins, respectively, were both upregulated at 2 DPI and 3 DPI (Fig. 3B).

### DEGs related to ROS accumulation and scavenging

**Fig. 4:**
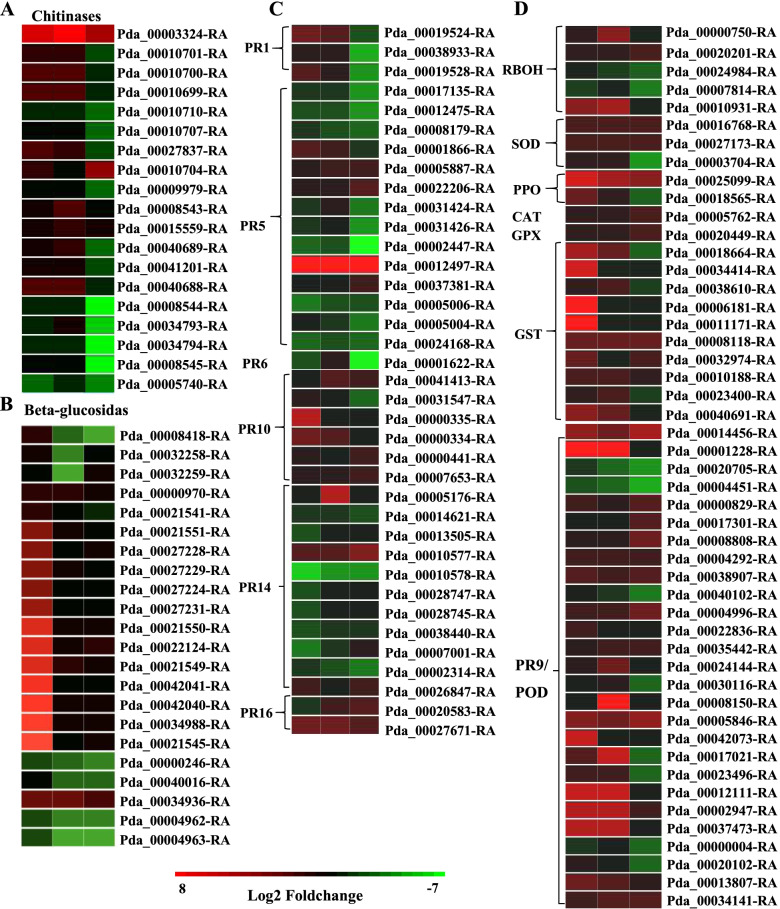
Heat maps of DEGs related to ROS signaling and pathogenesis-related proteins. The log_2_ fold change was colored using Cluster 3.0 (red for upregulated, green for downregulated), each horizontal row represents a DEG with its gene ID, and the vertical columns represent 2, 3, and 4 DPI from left to right

As the H_2_O_2_ content in leaves, as well as the enzyme activities involved in ROS scavenging, changed after infection, the DEGs related to ROS accumulation and scavenging were identified. Three genes encoding respiratory burst oxidase homolog (*RBOH*) proteins, which respond to pathogens by producing ROS, were upregulated during infection. Moreover, genes encoding enzymes involved in ROS scavenging also showed significant changes. The expression levels of genes such as *SOD*, *PPO*, *CAT*, *POD* and glutathione peroxidase (*GPX*) were mainly upregulated, especially for *PPO* and *POD*, which showed high expression during infection (Fig. 4D). In addition, the expression of glutathione S-transferases (*GSTs*) was mainly upregulated at 2 DPI, while only *GSTFB* (Pda_00008118-RA) was continuously activated during infection.

### Defense-related proteins

Pathogenesis-related (PR) proteins, which are indispensable components of plant innate immunity, play important roles in plant defense against pathogens. In this study, many poplar *PR* genes were induced in response to *A. alternata* infection; the expression of *PR-1* was upregulated at both 2 DPI and 3 DPI, but most of the thaumatin-like protein (*PR-5*) genes were downregulated, except one that had high transcript levels during pathogen infection (Fig. 4C). Our results also demonstrate that the *PR-9* s (peroxidases), *PR-10* s (ribonucleases), *PR-14 s* (lipid-transfer proteins) and *PR-16* s (germin-like proteins) were up- or downregulated throughout the infection period (Fig. 4C, D). Furthermore, most of the *chitinases* showed high transcript levels at both 2 DPI and 3 DPI but were downregulated at 4 DPI (Fig. 4A), while some of the *glucusidas* were upregulated at 2 DPI but then downregulated (Fig. 4B).

### DEGs related to plant hormone and signal transduction

**Fig. 5:**
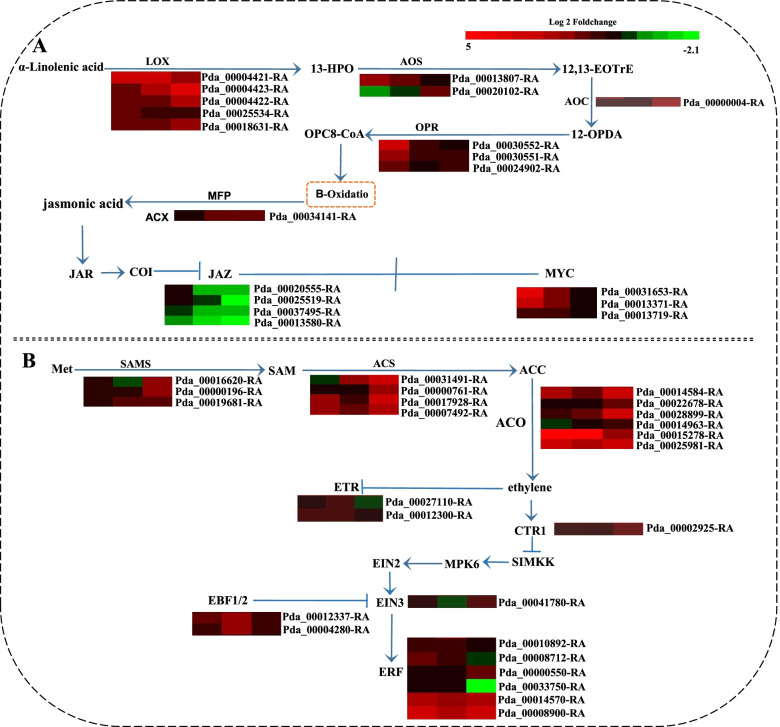
Expression patterns of genes related to JA/ET signaling during *A. alternata* infection. The log_2_ fold change was colored using Cluster 3.0 (red for upregulated, green for downregulated), each horizontal row represents a DEG with its gene ID, and the vertical columns represent 2, 3, and 4 DPI from left to right

Phytohormones, especially salicylic acid (SA), jasmonic acid (JA), and ethylene (ET), are critical regulators of plant-pathogen interactions; among them, the JA/ET signaling pathway is involved in the response to necrotrophic pathogen infection. In this study, DEGs related to JA/ET synthesis and signal transduction were identified, and most were upregulated during infection. Genes involved in JA synthesis, including linoleate lipoxygenase (*LOX*), allene oxide synthase (*AOS*), allene oxide cyclase (*AOC*), 12-oxophytodienoate reductase (*OPR*) and acyl-coenzyme oxidase (*ACX*), were induced during infection. The expression levels of *LOX*, *AOC*, *AOS3* (Pda_00013807-RA) and *ACX* were upregulated continuously, while the expression of *OPR3* (Pda_00030552-RA) was upregulated at 2 DPI but downregulated at 4 DPI. Moreover, the expression levels of *MYC*s involved in JA signal transduction were upregulated at both 2 DPI and 3 DPI. However, the expressions of JAZ, which contains a conserved TIFY domain and is a negative regulator of JA signal transduction, were mainly downregulated during pathogen infection (Fig. 5A). Similarly, genes for enzymes related to ET synthesis, including S-adenosylmethionine synthase (*SAMS*), 1-aminocyclopropane-1-carboxylate synthase (*ACS*) and 1-aminocyclopropane-1-carboxylate oxidase (*ACO*), were all upregulated during infection. Genes involved in the ET signal transduction pathway were also significantly induced. Five ethylene-responsive transcription factors *(ERFs*) were upregulated, while one *ERF* (Pda_00033750-RA) was downregulated. Among these *ERF*s, two *ERFC3*s (Pda_00014570-RA and Pda_00008900-RA) were highly induced at all time points (Fig. 5B). The SA response pathway has frequently been shown to be associated with plant resistance to biotrophic and hemibiotrophic pathogens. DEGs related to SA accumulation and signal transduction were also identified. The genes for enhanced disease susceptibility 1 (*EDS1*) and its coregulator phytoalexin deficient 4 (*PAD4*), which are related to SA accumulation, were both downregulated during infection. However, a few genes involved in the SA signal transduction pathway were induced. The *TGA1* gene (Pda_00012927-RA) was transcribed 1.03-fold at 2 DPI, and two *NPR* genes were upregulated at 4 DPI (Fig. S3)*.*

### The linoleate 13 -lipoxygenase gene (*PdbLOX2*) of *P*.* davidiana* × *P*.* bollena *is involved in resistance to *A*.* alternata*

**Fig. 6:**
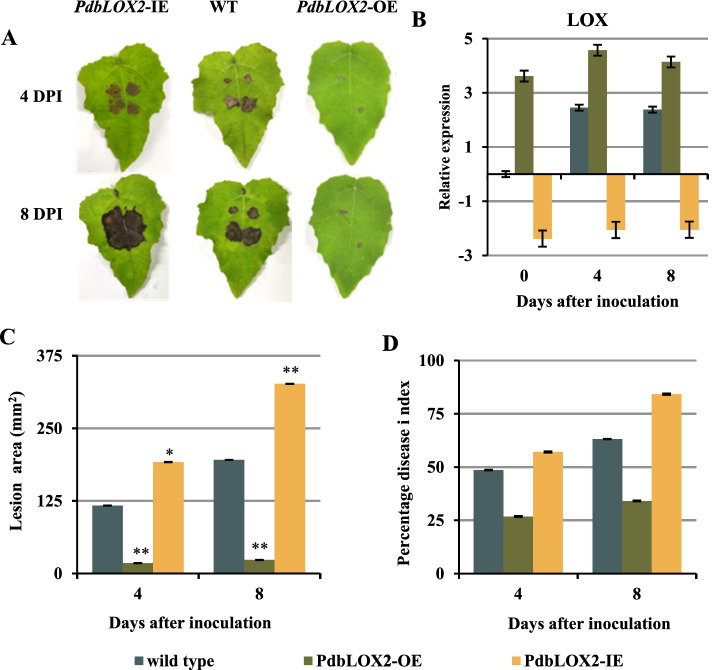
*PdbLOX2* enhance the resistance to *A. alternata* of poplar. **A** Leaves of WT and transgenic poplar inoculated with *A. alternata* plugs for 4 DPI and 8 DPI. **B** Relative expression level of *PdbLOX2* during infection in WT and transgenic plants. **C** Lesion area of leaves. **D** Percentage disease index at 4 and 8 DPI. The error bar represents the standard deviation of triplicate assays. Asterisks show significant differences compared with the corresponding controls. (**P < 0.01 and *P < 0. 05)

Lipoxygenase (LOX), a key enzyme in the JA biosynthetic pathway, was shown to be involved in plant defense responses against diverse pathogens. Based on the KEGG analyses and gene expression data, we found that several lipoxygenase genes were strongly induced during the infection of poplar leaves by *A. alternata*, especially *PdbLOX2* (Pda_00004421-RA); therefore, we speculated that this specific *LOX* gene plays an underlying role in regulating *P. davidiana* × *P. bollena* defense against *A. alternata* infection. The transgenic poplars with overexpressing *PdbLOX2* (*PdbLOX2*-OE) and RNAi-silenced *PdbLOX2* (*PdbLOX2*-IE) were obtained via Agrobacterium infection, and then the expressions of *PdbLOX2* were analyzed via RT-qPCR. *PdbLOX2* was expressed at significantly higher levels in *PdbLOX2-*OE lines than in the wild type (WT), but lower in *PdbLOX2*-IE lines (Fig. 6B). Next, leaves from transgenic poplars, as well as those from WT, were inoculated with *A. alternata*. After inoculation, the disease spots on WT leaves were dark, extensive, and irregular, while the disease spots on leaves of *PdbLOX2-*OE plants were light-colored circles, and were particularly smaller than WT. In contrast, the disease spots on leaves of *PdbLOX2-*IE plants were darker and larger than that on WT (Fig. 6A, C). All these results above were further confirmed by the percentage disease index (Fig. 6D).

**Fig. 7:**
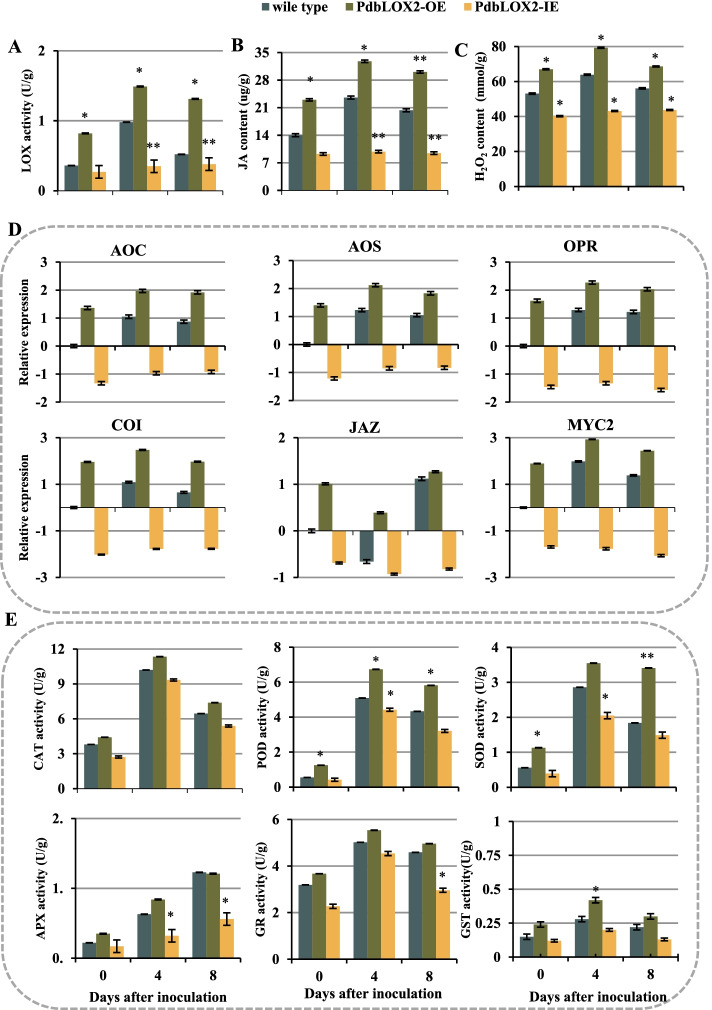
*PdbLOX2* involved in JA and ROS signals. **A** Activities of LOX in WT and transformed plants. **B** JA content in WT and over expressed plants. **C** Contents of H_2_O_2_ in WT and transformed plants. **D** Relative expression levels of JA biosynthetic and JA signaling pathway genes. **E** Activities of enzymes related to ROS scavenging. The error bar represents the standard deviation of triplicate assays. Asterisks show significant differences compared with the corresponding controls (**P < 0.01 and *P < 0. 05)

The lipoxygenase activity and jasmonic acid content were then analyzed in the transgenic poplars and WT during infection. The results showed that the lipoxygenase activity and the JA content in the overexpression lines were higher than those in the WT during infection, while the contents of two were lower in *PdbLOX2*-IE lines than in WT (Fig. 7A, B). In addition, in *PdbLOX2-*OE plants, JA-related genes, including *AOC*, *AOS*, *OPR*, *COI*, *JAZ* and *MYC*, showed significantly higher relative expression levels than in WT during infection but lower in *PdbLOX2*-IE plants (Fig. 7D). Lipoxygenase (LOX) is also involved in lipid peroxidation processes during the plant response to biotic and abiotic stresses. Compared to the WT plants, in *PdbLOX2*-overexpressing lines, the H_2_O_2_ accumulation was significantly induced, while it was inhibited in *PdbLOX2*-IE lines (Fig. 7C). Moreover, the antioxidant enzyme activities in the overexpression lines were higher than those in WT but lower in RNAi-silenced plants (Fig. 7E). These results implied that the *PdbLOX2* involved in the resistance of poplar to *A. alternate* infection by regulating the JA synthesis and signaling pathway as well as the ROS accumulation.

## Discussion

Recently, poplar leaf blight caused by *A. alternata* has become a common disease that has had a serious economic impact in China, but little is known about the molecular mechanisms underlying the resistance to *A. alternata* infection in poplar. In the present study, we investigated the mechanism of transcriptional response in *P. davidiana* × *P. bollena* caused by *A. alternata* infection. Through the result of transcriptomic analysis in both pathogen-infected (2 DPI, 3 DPI, 4 DPI) and mock-inoculated (0 DPI) leaves, 5,930 DEGs were identified, while 20.15% of them have no homologous genes after checking public databases, perhaps an indicator that those genes have yet to be identified in other plants. To determine the functional categories of the DEGs induced by pathogenic infection, GO and pathway enrichment analyses were performed. The results showed that most of the DEGs were involved in “plant hormone signal transduction” (ko04075) and “phenylpropanoid biosynthesis” (ko00940) or enriched for “defense response to fungus” and “plant-type cell wall”, which are all involved in plant defense against pathogens. These results are in line with plants potentially having evolved a series of molecular defense mechanisms during different infection stages of a pathogen.

### Phenylpropanoid compounds are involved in defense against *A. alternata* infection

Secondary metabolites formed through the phenylpropanoid biosynthetic pathway act in plant defenses ranging from preformed or inducible physical and chemical barriers to signaling molecules involved in local and systemic signaling to induce other defense genes [7, 36, 37]. Among these secondary metabolites, lignin has been shown to form a mechanical barrier when plants resist pathogen infection, and genes involved in catalyzing lignin, such as *PAL*, *HCT, CCR* and *CAD*, affect plant disease resistance [5, 38]*.* For example, in wheat, silencing genes encoding the monolignol biosynthesis enzymes, such as *PAL* and *CAD*, leads to susceptibility of leaf tissues to the fungal pathogen *Blumeria graminis* f. sp. *tritici* [39]*.* And in *Arabidopsis,* the *CAD* genes which have been demonstrated to be involved in lignin biosynthesis, act as essential components of defense against virulent and avirulent strains of the bacterial pathogen *P. syringae* pv. *tomato* [40]. The *PtHCT2* gene was shown to respond to infection by the pathogen *Sphaerulina musiva* in resistant *P. trichocarpa* cultivars compared to susceptible cultivars [41]*.* For our study, genes related to lignin biosynthesis in poplars, such as *PAL*, *4CL*, *HCT*, *CCR* and *CAD,* were induced during the interaction with *A. alternata* (Fig. 3A), which suggests that the lignin biosynthetic pathway responds to pathogen infection.

Moreover, flavonoids, including anthocyanins, flavonols, flavones, and proanthocyanidins, were also shown to play roles in protecting plants against pathogens. Genes related to the biosynthesis of these metabolites, especially *CHS* and *F3H,* were induced during pathogen infection. For example, the expression of *CHS* in soybean is upregulated during both fungal and bacterial pathogen infection [42]. Overexpression of *OxF3H* in rice increased tolerance to bacterial leaf blight [43]. In this study, DEGs related to flavonoid biosynthesis, including *CHS*, *F3H*, *DFR* and *LDOX,* were induced by *A. alternata* infection, especially at the early stage of infection (Fig. 3B), which indicated that poplar defends against *A. alternata* by inducing the production of flavonoids.

### ROS signaling is involved in defense against *A. alternata* infection

Pathogen infections induce the rapid accumulation of ROS, which limits the ingress of or causes oxidative damage to pathogens [44]. Simultaneously, excessive ROS can cause an increase in the levels of enzymatic and nonenzymatic antioxidants. The enzymes involved in oxidative bursts and scavenging, such as POD, GST, APX, SOD, and RBOH, are activated and induced by pathogen infection in plants. For example, genes encoding antioxidant and detoxifying enzymes, including *GST*s and *POD,* were significantly induced in the resistant cucumber genotype No. 26 but not in the susceptible genotype 26 M during *B. cinerea* infection [45]. Similarly, tomato bushy stunt virus infection significantly affects enzymes that are responsible for the balance of ROS accumulation, such as CAT and SOD, in *N. benthamiana* [46]. *Pyricularia oryzae* infection induces the expression of *OsRBOHB* and alters its localization in rice, which results in the focal accumulation of ROS around invasive hyphae and effectively inhibits pathogen infection [47]. In this study, the activity of enzymes involved in ROS scavenging, including SOD, POD, PPO and CAT, was notably induced during infection, which is in line with the expression patterns of the related genes. Other genes related to ROS production, such as *RBOH* and *LOX*, were also induced, while the H_2_O_2_ content significantly increased during infection (Fig. 1F-J, Fig. 4D). These results suggest that poplar may defend against infection by accumulating ROS.

### JA/ET signaling pathway activation after *A. alternata* infection

Plant responses to biotic stress are also coordinated by a network of signal transduction pathways that control a wide range of physiological processes. Commonly, the SA response pathway was shown to be associated with plant resistance to biotrophic and hemibiotrophic pathogens. In this study, most of genes related to SA signal were down-regulated. However, the *TGA1* and *NPR* were up-related during the infection (Fig. S3). Similar results were also observed in other transcriptome studies, such as chrysanthemum and pear interaction with *Alternaria* sp. [32, 48], *Physcomitrium patens,* tomato and rose interaction with *Botrytis cinerea* [49–51]*.* The results indicating that, *TGA* and *NPR* maybe involved in the other ways to defend against *A. alternata* infection in poplar. Moreover, JA/ET-dependent signaling is commonly known to be involved in basal or induced plant defense mechanisms against necrotrophic pathogens [9, 10]. The MYC protein, a positive regulator in the JA signaling pathway, was shown to play roles in plant defense by integrating signals from the JA and ET pathways. For example, MYC2 and its downstream MYC2-targeted TFs (MTFs) form a transcription module that may directly regulate the JA-induced transcription of late defense genes during tomato resistance to *B. cinerea* [52]. The overexpression of *OsMYC2* in rice resulted in the upregulation of early JA-responsive genes, as well as resistance to bacterial blight [53]. Moreover, numerous *ERF* genes have been shown to be involved in JA-mediated defense responses; these genes include *AtERFs* in *Arabidopsis* [54–58], *TaERF3* and *TaPIEP1* in wheat [59, 60], and *OsERF*s and *OsEREBP1* in rice [61–63]. In this study, genes involved in JA/ET signaling and biosynthesis, such as *LOX*, *AOS*, *MYC*, and *ERF* were induced, and the JA content significantly increased during infection (Fig. 1, Fig. 5)*.* These results suggested that *P. davidiana* × *P. bollena* may resist *A. alternata* infection mainly via the JA/ET signaling pathway.

### *PdbLOX2* participates in poplar resistance and defense by mediating JA signaling pathways and ROS production

Lipoxygenase plays roles in plant resistance, including initiating the synthesis of signaling molecules, such as JA or MeJA, or changing the metabolism in the cell, which leads to a high ROS content and ultimately causes cell death [64–68]. For example, the early activation of *LOX* genes and other JA biosynthetic genes was shown to be involved in resistance in maize, and *ZmLOX3* in maize may be involved in fungal pathogenesis by mediating oxylipin metabolism; the loss of *ZmLOX3* function leads to susceptibility to *Aspergillus flavus* and *A. nidulans* infection in maize [69, 70]. In cotton, *GhLOX2* positively regulates tolerance to *Verticillium dahliae* infection via JA signaling, while silencing *GhLOX2* inhibited H_2_O_2_ accumulation, which impaired cotton resistance [71]. In this study, we obtained transgenic poplar plants that overexpressed *PdbLOX2* as well as the RNAi plants*.* The *PdbLOX2*-OE plants were more resistant to *A*. *alternata* infection than WT plants, whereas *PdbLOX2*-IE lines showed reduced resistance to *A*. *alternata* compared to WT (Fig. 6)*.* Moreover, the overexpression of *PdbLOX2* increased the transcription of JA-signaling-related genes and promoted JA accumulation. In addition, the H_2_O_2_ content increased significantly, and ROS-scavenging enzymes were activated in *PdbLOX2*-OE lines. While the opposite physiological changes were observed in *PdbLOX2*-IE plants (Fig. 7). Overall, these results suggest that the *PdbLOX2* is involved in the resistance of poplar to *A. alternate* infection by regulating the JA synthesis and signaling pathway as well as the ROS accumulation.

## Conclusions

**Fig. 8:**
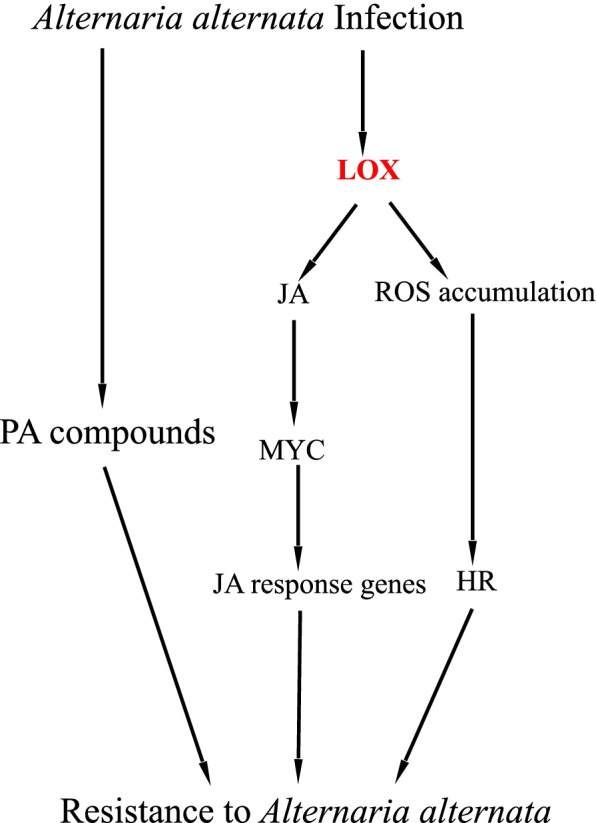
Potential mechanism of poplar resistance to *A. alternata*

In summary, we used RNA-Seq technology to perform a global transcription analysis of *P. davidiana* × *P. bollena* in response to *A. alternata* infection, and the lipoxygenase gene *PdbLOX2* was selected for further study; that analysis helped us better understand the transcriptional response of poplar to *A. alternata* infection and in the future breed durable, broad-spectrum disease-resistant crops. A total of 5,930 DEGs were obtained via RNA-Seq, and functional annotation showed that a variety of defense responses mediated by ROS accumulation, phenylpropanoid compound (PA compounds) production and JA/ET signaling were activated. Then, the *PdbLOX2* was shown to be involved in the resistance of poplar to *A. alternate* infection by regulating the JA synthesis and signaling pathway as well as the ROS accumulation by genetic engineering. Our results suggest that JA/ET signaling, the ROS metabolic pathway and PA compounds, which constitute a coordinated defense network, are involved in the response of poplar to *A. alternata* infection (Fig. 8).

## Methods

### Plant materials, pathogen culture, and inoculation method

The phytopathogenic fungus *Alternaria alternata* was cultured for 10 days on potato dextrose agar (PDA) medium in the dark and then diluted with sterile water to 1 × 10^6^ spores/mL. Seedlings of *Populus davidiana* × *P. bollena* were grown in a greenhouse under 16-h light/8-h dark conditions at 25 °C for three months. Then, healthy leaves were inoculated with 10 μl of spore suspension and leaves without inoculation served as the mock control (0 DPI). The experimental design followed the method of Yang. [72]. All treatments applied are initiated at different calculated times prior to the harvest day, and all samples are designed to be harvested at the same time. The leaves were observed for symptom development every day after inoculation. Disease ratings and biochemical assays were performed at 2, 3, and 4 d after inoculation (DPI). Leaf lesion areas were measured by ImageJ software [73, 74]. The infection ratings were assigned and calculated according to the method of Pandey [75]. Three biological replicates, each containing ten parallel leaves from ten different plants, were used for each treatment time point or control. The samples were then placed in liquid nitrogen rapidly and stored at − 70 °C.

### Biochemical assays

The samples were used to determine the contents of phytohormones, H_2_O_2_ and the activity of enzymes, including peroxidase (POD), polyphenol oxidase (PPO), superoxide dismutase (SOD), catalase (CAT) and phenylalanine ammonia-lyase (PAL). The phytohormones contents, including SA and JA were determined via enzyme-linked immunosorbent assay (ELISA) kits (Shanghai, China BYE97056 and RJ-21830, respectively), and the H_2_O_2_ content was determined via a hydrogen peroxide assay (Nanjing, China A064). The activities of PAL, SOD, POD as well as CAT were measured based on the methods of Liu [76]. The PPO activity was measured according to the method of Hammerschmidt [77]. For each assay, three biological replicates were used.

### RNA extraction, library construction and sequencing

Total RNA was isolated using an RNeasy Plant Mini Kit and treated with RNase-free DNase I (Qiagen, Hilden, Germany). The quality of the RNA was checked via agarose gel electrophoresis to ensure that clear bands were visible. The integrity and quantity of the RNA samples were determined using a NanoDrop2000 spectrophotometer (Thermo Fisher Scientific, Wilmington, DE, USA) and an Agilent Bioanalyzer 2100 (Agilent Technologies, Santa Clara, CA, USA). RNA-Seq cDNA libraries were constructed from mRNA using an NEB Next UltraTM RNA Library Prep Kit for Illumina (NEB, USA) according to the manufacturer’s instructions. Finally, twelve cDNA libraries (three at  0 DPI, three at 2 DPI, three at 3 DPI and three at 4 DPI) were constructed for sequencing. RNA-Seq was performed on the Illumina HiSeq 2500 platform to obtain paired-end reads. The RNA-Seq data were deposited in the NCBI Sequence Read Archive database (http://www.ncbi.nlm.nih.gov/sra/) under project accession number PRJNA649854.

### Analysis of differentially expressed genes and functional annotation

To obtain clean reads, the adaptor reads, low-quality reads (Q-value < 20) and reads those containing more than 10% ambiguous “N” bases were discarded. Clean reads were mapped to the *Populus davidiana* genome (GenBank: GCA_014885075.1) using Tophat2 software with default parameter settings [78]. To identify DEGs in poplar in response to *A*. *alternata* infection at different time points after inoculation, the expression level of each transcript was calculated based on the FPKM (fragments per kilobase of exons per million mapped reads) with the software package Cufflinks [79]. Differential expression analysis of the two samples was performed using the DESeq2 R package. | log_2_fold change|≥ 1 and false discovery rate (FDR) values < 0.05 were used as cutoff criteria for DEGs. Gene Ontology (GO) enrichment analysis and Kyoto Encyclopedia of Genes and Genomes (KEGG) pathway analysis of the DEGs were performed using eggNOG-mapper software [80].

### Real-time quantitative PCR validation of expression profiles

Total RNA was extracted from the leaves of 3-month-old plants and transgenic plants treated with *A. alternata* for 0 (control), 2, 3 and 4 d using an RNeasy Plant Mini Kit (Qiagen, Hilden, Germany), and cDNA was synthesized for quantitative real-time PCR (RT-qPCR) using the TransScript One-Step gDNA Removal and cDNA Synthesis SuperMix Kit (AT311, TransGen Biotech, Beijing, China) according to the manufacturer’s method. RT-qPCR was conducted using SYBR® Green Real-time PCR Master Mix (QPK-201, Toyobo, Osaka, Japan) in a CFX96 TouchTM Real-Time PCR detection system (Bio–Rad, Hercules, CA, USA). Three biological replicates, each with three technical replicates, were used per sample. The housekeeping genes *PdbEF* and *Pdbactin* were selected as the internal controls, and the primers used for RT-qPCR analysis are listed in Table S3. Relative gene expression levels were measured using the 2^−ΔΔCt^ method [81].

### Generation of transgenic plants

The complete ORF of *PdbLOX2* was cloned into the *KpnI* and *XbaI* restriction enzyme sites of pROKII under the control of the CaMV 35S promoter and NOS terminator to generate the overexpression construct pROKII-*PdbLOX2*. And a 203 bp truncated inverted repeat CDS of *PdbLOX2* was inserted into RNAi vector pFGC5941 (*pFGC*::*PdbLOX2)* to silence the expression of *PdbLOX2*. Then, the constructs were introduced into Agrobacterium strain EHA105, which were then transformed into *P. davidiana* × *P. bollena* via *Agrobacterium*-mediated leaf-disk transformation, respectively. The transgenic poplars were selected using kanamycin (50 mg/L) for over expression plants (*PdbLOX2*-OE), or glyphosate (1 mg/L) for RNA silenced plants (*PdbLOX2*-IE), and all transgenic poplars were verified via genomic PCR and RT-qPCR. The primers used for transformation are listed in Table S3.

### *Alternaria alternata* resistance assay in transgenic plants

Leaves collected from three-month-old transgenic plants and wild type plants were inoculated with *A. alternata* plugs (5 mm) and then placed in a transparent square Petri dish under the conditions described above. The leaves were observed and sampled 4 d and 8 d after inoculation. Pathogen resistance assays were repeated in three independent experiments. Each independent experiment included three biological replicates, each with three technical replicates, and leaves from at least 9 individual plants were pooled together as one biological replicate. The sampled leaves were used to determine the JA and H_2_O_2_ contents; the activity of enzymes, including lipoxygenase (LOX), POD, CAT, SOD, ascorbate peroxidase (APX) and glycopeptide reductase (GR); and RT-qPCR. The contents of JA and H_2_O_2_ were determined as described above. The activities of CAT, SOD, POD and APX were measured as described above. The determination and calculation of LOX activity were performed using a lipoxygenase (LOX) activity assay kit (BC0320, Beijing Solarbio Science & Technology, Beijing, China). GR activity was measured as described by Ge [82]. GST activity was measured as described by Horváth [83]. RT-qPCR followed the method described above, and the primers used for analysis are listed in Table S3. For each assay, three biological replicates were analyzed.

### Statistical analyses

Statistical analyses were carried out using SPSS v.20 software. The data were compared using Student’s t-test. Differences between two groups of data were considered statistically significant at *p* < 0.05. * means significant difference.

## Supplementary Information

**Additional file 1: Fig. S1.** Validation of RNA-Seq Data by RT–qPCR.

**Additional file 2: Fig. S2.** Heat maps of DEGs related to defense.

**Additional file 3: Fig. S3.** Heat maps of DEGs involved in SA signal transduction.

**Additional file 4: Supplementary Table S1.** Summary of RNA-seq data from 12 samples.

**Additional file 5: Supplementary Table S2.** DEGs between samples.

**Additional file 6: Supplementary Table S3.** Primers sequences used in this study.

## Data Availability

The data used to support the findings of this study are available from the corresponding author upon request. The data from this study are available from the NCBI Sequence Read Archive database under accession number PRJNA649854.
