# Supplementary material for: Integrated transcriptomic and transgenic analyses reveal potential mechanisms of poplar resistance to Alternaria alternata infection
Source: BMC Plant Biol. 2022 Aug 25;22:413. doi: 10.1186/s12870-022-03793-5 (PMC9404672; doi:10.1186/s12870-022-03793-5)
Supplement: Supplementary file 1 — Additional file 1: Fig. S1. Validation of RNA-Seq Data by RT–qPCR. [file 12870_2022_3793_MOESM1_ESM.pdf]

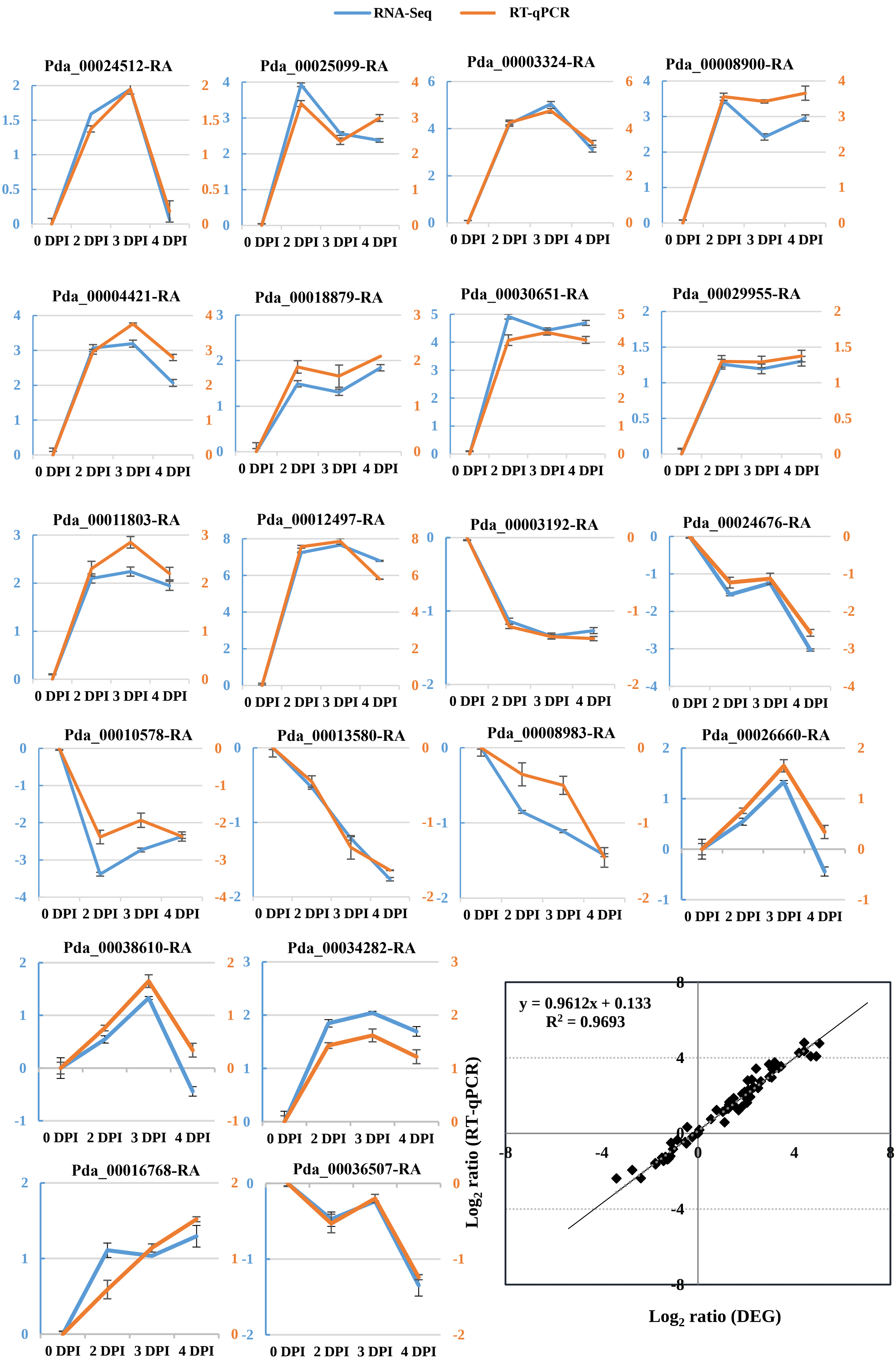

RNA-Seq
RT-qPCR
Pda_00024512-RA
Pda_00025099-RA
Pda_00003324-RA
Pda_00008900-RA
Pda_00004421-RA
Pda_00018879-RA
Pda_00030651-RA
Pda_00029955-RA
Pda_00011803-RA
Pda_00012497-RA
Pda_00003192-RA
Pda_00024676-RA
Pda_00010578-RA
Pda_00013580-RA
Pda_00008983-RA
Pda_00026660-RA
Pda_00038610-RA
Pda_00034282-RA
Pda_00016768-RA
Pda_00036507-RA
0 DPI 2 DPI 3 DPI 4 DPI
y = 0.9612x + 0.133
R^2 = 0.9693
Log$_2$ ratio (RT-qPCR)
Log$_2$ ratio (DEG)

Fig. S1 Validation of RNA-Seq Data by RT–qPCR

The left y-axes represent the gene expression levels determined by RNA-seq. The right y-axes represent the gene expression levels determined by RT–qPCR. The relative expression levels are shown as the $\log_2$ transformed fold change (FC) values. Correlation: correlation analysis of the results between real-time RT-qPCR and RNA-Seq, and correlation coefficient R^2 is 0.969 ($P < 0.05$)
