# Supplementary material for: Integrated transcriptomic and transgenic analyses reveal potential mechanisms of poplar resistance to Alternaria alternata infection
Source: BMC Plant Biol. 2022 Aug 25;22:413. doi: 10.1186/s12870-022-03793-5 (PMC9404672; doi:10.1186/s12870-022-03793-5)
Supplement: Supplementary file 3 — Additional file 3: Fig. S3. Heat maps of DEGs involved in SA signal transduction. [file 12870_2022_3793_MOESM3_ESM.pdf]

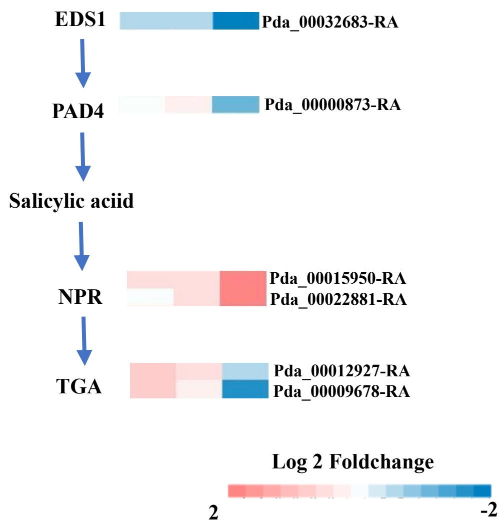

Fig. S3 Heat maps of DEGs involved in SA signal transduction

The $\log_2$ fold change was colored using Cluster 3.0 (red for upregulated, blue for downregulated), each horizontal row represents a DEG with its gene ID, and the vertical columns represent 2, 3, and 4 DPI from left to right.
