# Supplementary material for: Integrated transcriptomic and transgenic analyses reveal potential mechanisms of poplar resistance to Alternaria alternata infection
Source: BMC Plant Biol. 2022 Aug 25;22:413. doi: 10.1186/s12870-022-03793-5 (PMC9404672; doi:10.1186/s12870-022-03793-5)
Supplement: Supplementary file 4 — Additional file 4: Supplementary Table S1. Summary of RNA-seq data from 12 samples. [file 12870_2022_3793_MOESM4_ESM.docx]

**Supplementary Table S1** **Summary of RNA-seq data from 12 samples**

| Sample | Raw reads | Clean reads | Mapped reads | Mapped rates (%) | Clean bases (G) | Q20 (%) | Q30 (%) | GC% |
| --- | --- | --- | --- | --- | --- | --- | --- | --- |
| 0 DPI-1 | 40,500,301 | 40,021,410 | 28,783,404 | 70.48 | 7.47 | 97.10 | 92.11 | 44.33 |
| 0 DPI-2 | 45,201,042 | 44,464,280 | 30,538,646 | 70.94 | 7.51 | 97.23 | 93.04 | 44.10 |
| 0 DPI-3 | 43,012,350 | 42,141,266 | 29,940,598 | 70.64 | 7.23 | 97.30 | 92.76 | 44.16 |
| 2 DPI-1 | 45,341,606 | 44,448,940 | 34,146,462 | 71.11 | 7.50 | 97.13 | 91.86 | 44.11 |
| 2 DPI-2 | 47,514,200 | 47,011,078 | 35,219,372 | 71.65 | 7.55 | 97.26 | 92.43 | 44.17 |
| 2 DPI-3 | 49,035,418 | 48,175,320 | 36,779,165 | 72.24 | 7.58 | 97.01 | 92.01 | 44.08 |
| 3 DPI-1 | 52,429,670 | 51,931,021 | 38,503,078 | 73.68 | 7.60 | 97.29 | 92.22 | 43.87 |
| 3 DPI-2 | 53,662,930 | 53,010,158 | 38,902,614 | 72.79 | 7.61 | 97.11 | 91.96 | 44.01 |
| 3 DPI-3 | 54,118,182 | 53,500,910 | 39,240,428 | 73.12 | 7.88 | 97.17 | 92.11 | 43.93 |
| 4 DPI-1 | 53,041,404 | 52,753,410 | 37,811,272 | 72.66 | 7.60 | 97.07 | 92.05 | 44.06 |
| 4 DPI-2 | 54,141,024 | 53,716,462 | 38,659,410 | 73.39 | 8.03 | 97.11 | 92.01 | 44.16 |
| 4 DPI-3 | 50,966,740 | 50,175,182 | 36,669,079 | 72.87 | 7.59 | 97.20 | 92.15 | 44.11 |
| Average | 49,080,405 | 48,445,786 | 35,432,794 | 72.13 | 7.60 | 97.17 | 92.23 | 44.09 |
