# Supplementary material for: Integrated transcriptomic and transgenic analyses reveal potential mechanisms of poplar resistance to Alternaria alternata infection
Source: BMC Plant Biol. 2022 Aug 25;22:413. doi: 10.1186/s12870-022-03793-5 (PMC9404672; doi:10.1186/s12870-022-03793-5)
Supplement: Supplementary file 6 — Additional file 6: Supplementary Table S3. Primers sequences used in this study. [file 12870_2022_3793_MOESM6_ESM.pdf]

Supplementary Table S3 Primers used for RT-qPCR

Gene ID	Functional annotation	Forward primers(5'-3')	Reverse primers(5'-3')
Pda_00024512-RA	Disease resistance protein RPM1	GGAAGAGGTTTAATGGATCACCCTCG	GTGCTCTTTCACCCTGTGGCTGTCAT
Pda_00025099-RA	Polyphenol oxidase	CTGCTGCTCTCCATTACCCAGAAAGAT	GGCACAATGGACATTGGCTTGTTGGT
Pda_00003324-RA	Chitinase-like protein 2	GACAATGGCGTTGCTAGTAATGCTAG	GTGTTACGTTTCGAAAACAAGTTTTCG
Pda_00008900-RA	Ethylene-response factor C3	TGTGGTGGCACTGAAGAAGAAACACTC	GGAGATGATGGCGAGAATTCTAGATTT
Pda_00004421-RA	Linoleate 13S-lipoxygenase 2-1	TGGCAGTGGTCATGCTTCTTTTCGTG	CAAGTCCTCGTTCTATCCCTATATCT
Pda_00018879-RA	Transcription factor bHLH87	GAACAGAGATTGTTCGCAAACTAAGC	GATGAGCTTGGACGCTTATCTGATCT
Pda_00030651-RA	NAC domain-containing protein 43	CATCAAGCTTATTGGAGGCTCATCAG	GGTGCATGAATCTTTCATGGAGCCCAT
Pda_00029955-RA	Putative disease resistance protein RGA3	ATCCTTAAAGAGACTTCGTATCTTTAG	ACTAGAGAAGGCCAATTGAAGACTTTC
Pda_00011803-RA	Homeobox-leucine zipper proteinATHB-17	TGTAATGACAGAAAACACCAGGCTCG	CTACAGGAAAGATTTCATCATTTCCAG
Pda_00012497-RA	Thaumatin-like protein	CTTGACATGCCATTCTTTGCTATCAGGG	TCGAGCCCACATTCGGCCATTCCAATC
Pda_00003192-RA	Transcription factor HHO2	ATGGACAATCGGAATGCTCTGAGCAG	GCTATTGAACTAGTTGCAGAAGCCGG
Pda_00024676-RA	Zinc transporter 8	ACCTTCATTTGCTTGCTTCTACTACTCC	AGTCTTTTCAGGGCGTAAAACGGGGAT
Pda_00010578-RA	Non-specific lipid-transfer protein	TGGTGCTCTCCGCACCCATGTTGAAC	GGCGGTTGTTTTAATGCAATTACAAG
Pda_00013580-RA	Protein TIFY 9	CCTGCTTTGCCTGTCTATTTCCCTCT	GTTGGTGATCTGTTCTTGGATCGGGT
Pda_00008983-RA	Annexin D4	ATGGTAACAAGAAGAATCCCATTGAGG	CCAAAACCTTGCTGAAGTATGCATGAG
Pda_00000004-RA	Allene oxide cyclase	CCCAAGATCCCAATCTCTCTTCCAATC	GGTTTTGGAGATACTGGAGGGGACC
Pda_00013807-RA	Allene oxide synthase 3	CTGAATCAAAACTCCCGATGAAG	GCATCAAGGACAGCAATGACTT
Pda_00030552-RA	12-oxophytodienoate reductase 2	ATGGATGCTGTGACTCCCACGATG	CTTGTGCTGTGTCAGAAACTCCAG
Pda_00006471-RA	Coronatine-insensitive protein 1	ACCTTGAGCTTCTTGCCAGGAAC	TCTCCATGTAGCTAAGACCCAAAC
Pda_00031653-RA	Transcription factor MYC2	TTTGGGTCAGATCTTAGCGCTTATT	CAAATAAGACAGGCCTGGTTTATT
Pda_00026660-RA	Transcription factor WRKY 27	GCCAGTGGACTTCAAGGCAATAAC	GATTTTGTTGTTGCTGTAGAATATG
Pda_00038610-RA	Glutathione S-transferase	AGGTGCTTTACTTCTTCAAATGAATC	GCTTGAGGCTGTCAATGAAATCC
Pda_00034282-RA	Transcriptional factor MYB	CTGATAATTCAAGCACGACCAC	:CCCATGGAGAGTTACTATCCTC
Pda_00016768-RA	superoxide dismutase [Cu-Zn]	TCGCTGCCAAGAAGCAACCACCTC	GGAATCCATGAGGCCCTGGAG
Pda_00036507-RA	Transcription factor bHLH153	TAAGGGATCGAAGTGTTTGAAG	CTCCATAAGGACAGATGCAGTA
KP973951	PdbEF1-α	TGGGTCGTGTTGAAACTGGTGT	GGCAGGATCGTCCTTGGAGTTC
KP973950	Pdbactin	GCTGAGAGATTCCGTTGCCCTG	GGCGGTGATCTCCTTGCTCATT

Primers used for transformation

	Forward primers(5'-3')	Reverse primers(5'-3')
pROKII-*PdbLOX*	AGTCGGTACCATGTTGAAGCCACAGCTTCACCAG	GCATTCTAGATCAGATAGAGATGCTATAAGGAAC
pFGC5941-*PdbLOX*-Cis	ATCGCCAATGGTGGCAGTGGTCATGCTTCTTTTCGTG	ATCGGGCGCGCCAAGTCCTCGTTCTATCCCTATATCT
pFGC5941-*PdbLOX*-Anti	ATCGTCTAGATGGCAGTGGTCATGCTTCTTTTCGTG	ATCGGGATCCCAAGTCCTCGTTCTATCCCTATATCT
